# IgIDivA: immunoglobulin intraclonal diversification analysis

**DOI:** 10.1093/bib/bbac349

**Published:** 2022-08-30

**Authors:** Laura Zaragoza-Infante, Valentin Junet, Nikos Pechlivanis, Styliani-Christina Fragkouli, Serovpe Amprachamian, Triantafyllia Koletsa, Anastasia Chatzidimitriou, Maria Papaioannou, Kostas Stamatopoulos, Andreas Agathangelidis, Fotis Psomopoulos

**Affiliations:** Institute of Applied Biosciences, Centre for Research and Technology Hellas, Thessaloniki, Greece; Hematology Unit, 1st Dept of Internal Medicine, Aristotle University of Thessaloniki, AHEPA Hospital, Thessaloniki; Anaxomics Biotech SL, Barcelona, Spain; Institute of Biotechnology and Biomedicine, Universitat Autònoma de Barcelona, Barcelona, Spain; Institute of Applied Biosciences, Centre for Research and Technology Hellas, Thessaloniki, Greece; Institute of Applied Biosciences, Centre for Research and Technology Hellas, Thessaloniki, Greece; Institute of Applied Biosciences, Centre for Research and Technology Hellas, Thessaloniki, Greece; Department of Pathology, School of Medicine, Aristotle University of Thessaloniki; Institute of Applied Biosciences, Centre for Research and Technology Hellas, Thessaloniki, Greece; Department of Molecular Medicine and Surgery, Karolinska Institute, Stockholm, Sweden; Hematology Unit, 1st Dept of Internal Medicine, Aristotle University of Thessaloniki, AHEPA Hospital, Thessaloniki; Institute of Applied Biosciences, Centre for Research and Technology Hellas, Thessaloniki, Greece; Department of Molecular Medicine and Surgery, Karolinska Institute, Stockholm, Sweden; Institute of Applied Biosciences, Centre for Research and Technology Hellas, Thessaloniki, Greece; Faculty of Biology, National and Kapodistrian University of Athens, Athens, Greece; Institute of Applied Biosciences, Centre for Research and Technology Hellas, Thessaloniki, Greece

**Keywords:** intraclonal diversification, B cell receptor immunoglobulin, high-throughput sequencing, graph networks, graph metrics

## Abstract

Intraclonal diversification (ID) within the immunoglobulin (IG) genes expressed by B cell clones arises due to ongoing somatic hypermutation (SHM) in a context of continuous interactions with antigen(s). Defining the nature and order of appearance of SHMs in the IG genes can assist in improved understanding of the ID process, shedding light into the ontogeny and evolution of B cell clones in health and disease. Such endeavor is empowered thanks to the introduction of high-throughput sequencing in the study of IG gene repertoires. However, few existing tools allow the identification, quantification and characterization of SHMs related to ID, all of which have limitations in their analysis, highlighting the need for developing a purpose-built tool for the comprehensive analysis of the ID process. In this work, we present the immunoglobulin intraclonal diversification analysis (IgIDivA) tool, a novel methodology for the in-depth qualitative and quantitative analysis of the ID process from high-throughput sequencing data. IgIDivA identifies and characterizes SHMs that occur within the variable domain of the rearranged IG genes and studies in detail the connections between identified SHMs, establishing mutational pathways. Moreover, it combines established and new graph-based metrics for the objective determination of ID level, combined with statistical analysis for the comparison of ID level features for different groups of samples. Of importance, IgIDivA also provides detailed visualizations of ID through the generation of purpose-built graph networks. Beyond the method design, IgIDivA has been also implemented as an R Shiny web application. IgIDivA is freely available at https://bio.tools/igidiva

## Introduction

Recognizing antigens is B cells’ ‘raison d’être’. This is accomplished through the immunoglobulin (IG), which forms the part of the B cell receptor (BcR) that mediates antigen recognition [1, 2]. Considering the enormous antigen diversity in nature, it is obvious that a correspondingly vast repertoire of antigen-specific B cells with diverse BcR IG is warranted to endow the host with immune competence. The extraordinary diversity of the human BcR IG repertoire relies largely on V(D)J recombination, a combinatorial association of distinct IG heavy and light chain variable (V), diversity (D; for heavy chains only) and joining (J) genes occurring in developing B cells. Moreover, the variable regions of the IG heavy and light chains, representing the antigen binding sites, comprise four framework regions (FR) and three hypervariable complementarity determining regions [3]. Successful completion of V(D)J recombination leads to the expression of functional BcR IG of both IgM and IgD isotypes on the surface of naive B cells, rendering them competent to effectively recognize antigens [2, 4]. Once this happens, B cells mature further in specific microenvironments within the secondary lymphoid organs, called germinal centers, through two distinct molecular processes: somatic hypermutation (SHM) and class-switch recombination (CSR) [1, 5, 6]. Both processes are catalyzed by the enzyme activation-induced deaminase (AID) [7, 8]. SHM mostly entails the introduction of point mutations in the IG variable domain. These mutations can alter the affinity of the antibody for its cognate antigen, with mutations that lead to an increase in affinity being promoted [9]. The introduction of mutations within rearranged genes occurs at rates of 10^−5^–10^−3^ mutations per base pair per generation, 10^6^-fold higher than spontaneous mutations occurring elsewhere in the genome [10, 11]. On the other hand, CSR is responsible for the replacement of the IG heavy chain constant gene from IGHM/IGHD to IGHG or IGHE or IGHA, switching antibody production from IgM/IgD to a different class, such as IgG, IgE or IgA, without altering the antigen specificity of the antibody [12].

The aforementioned BcR IG diversity of the immune system in a healthy individual is reflected in the polyclonality of the respective repertoire. Human diseases implicating B cells may vary in terms of BcR IG gene repertoire diversity: some are polyclonal (for instance, systemic lupus erythematosus is associated with intense polyclonal B cell activation) [13], whereas others are characterized by oligoclonal (e.g. rheumatoid arthritis and multiple sclerosis) [14, 15] or even monoclonal B cell expansions (B lymphoid malignancies) [16].

An additional level of complexity may arise when focusing on specific, relevant B cell clonal expansions. In such a context, BcR IG repertoire diversity may increase through a process known as intraclonal diversification (ID), which entails the introduction of ongoing SHMs due to continuous antigenic pressure [6, 17, 18]. Studies of the ID process have provided valuable insight into the ontogeny and evolution of B cell clones in health and disease [19–23]. However, most relevant studies were performed using low-throughput, Sanger sequencing; hence, they were inherently limited with regard to analytical depth and breadth [22–28]. This limitation was recently surpassed due to the advent of next-generation sequencing (NGS), allowing a deeper and, thus, more accurate capture of the diversity of the BcR IG gene repertoire, both at the clonal and the subclonal levels, the latter being directly associated with ID [29–31]. However, in order to fully understand the complex immunogenetic ‘mechanics’ of ID, purpose-built bioinformatic tools are required.

Currently, several different bioinformatic approaches exist for the analysis and visualization of SHM within the BcR IG gene rearrangement sequences and their classification in the context of ID, such as ClonalTREE [32], GCTree [33], GLaMST [34], IgTree [35], MTree [36], ViCloD [37], Alakazam [38, 39] and AncesTree [40]. While ClonalTREE was developed for a different purpose (bacterial composition evolution), the rest are dedicated to the study of BcR IG repertoires. That notwithstanding, most existing solutions display one or more of the following limitations: (i) the analytical process in all of them is based on the inference of mutational variants, relying on statistical analysis for network generation, which can lead to arbitrary results and overcomplicated graphs; (ii) BcR IG sequence classification is based on phylogenetic tree-constructing programs, which is not ideal, given the high level of identity between the different sequences, often differing in only a few mutations, and given that traditional phylogenetic analysis are not suitable for these cases [41, 42]; (iii) they do not provide any analysis of graph metrics that could greatly assist in the quantification of ID levels and, subsequently, in performing comparisons between samples or groups of samples; (iv) some of them are not publicly available; (v) they are restricted to the analysis of the IG heavy chain gene repertoire; (vi) they do not provide a user-friendly interface that would enable their application without prior programming knowledge and (vii) they are visualization-only tools.

Here, we present the immunoglobulin intraclonal diversification analysis (IgIDivA) tool, a purpose-built tool for the detailed assessment of the ID process through the analysis of high-throughput NGS data. IgIDivA provides a detailed characterization of all mutations occurring in the context of the ID process as well as their connections. Subsequently, connections are used for ‘building’ mutational pathways, while a series of graph metrics are calculated toward a multifaceted characterization of ID. Finally, statistical analysis is provided for comparisons of different features of ID among samples or groups of samples. Overall, IgIDivA is a user-friendly pipeline, also available as an R Shiny app, freely available at https://bio.tools/igidiva.

## Materials and methods

### Data pre-processing

**Figure 1:**
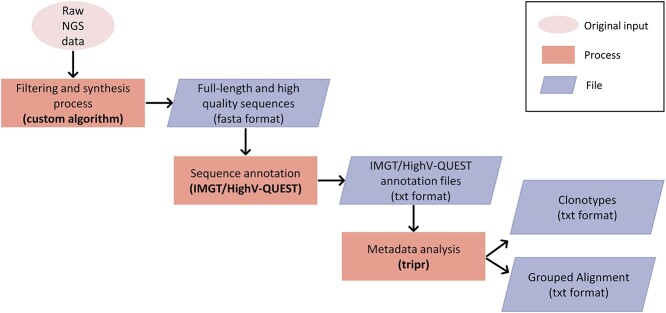
Workflow of the pre-processing of the data before the use of IgIDivA. The main steps are represented—from the obtention of the NGS raw data to the obtention of the two files (clonotypes computation and grouped alignment) that are used as input for IgIDivA. The original input is represented as an oval. The processes are represented as rectangles and the files are represented as parallelograms. The output files of the processes are the input files of the following ones.

The main pre-processing workflow, depicted in Figure 1, consists of (i) quality filtering and synthesis of raw reads (in the case of paired-end sequencing) in order to obtain high-quality, full-length BcR IG sequences; (ii) data annotation with IMGT/HighV-QUEST [43] and (iii) meta-data analysis with tripr (T cell receptor/immunoglobulin profiler in R) for the grouping of sequences into clonotypes and their alignment [44]. IgIDivA requires as input two tripr output files per sample. First, the ‘clonotypes computation’ file, i.e. grouping of BcR IG gene rearrangement sequences into clonotypes. Second, the ‘grouped alignment’ file, which concerns the alignment of all BcR IG gene rearrangement sequences against the respective germline V genes at the nucleotide (nt) level. Each unique sequence or group of identical sequences is considered as a nucleotide variant (nt var). The combination of these two files allows for the subsequent selection of specific clonotypes.

### Processing of the input

The IgIDivA approach is based on the assumption that, during the process of ID, SHMs are acquired sequentially due to continuous interactions with antigen(s). Hence, identifying the ‘timeline’ of SHM is a critical aspect of the process.

The first step in our approach concerns clonotype selection: depending on the context, the simplest option would be to focus on the most expanded (i.e. the most frequent) clonotype. However, any number of clonotypes can be selected per sample and each clonotype will be processed independently.

Subsequently, the ‘grouped alignment’ file should be filtered in order to limit the analysis to the selected clonotype(s).

### Network generation

Network generation comprises the identification of all different nt vars, their SHM patterns and their connections.

First, the main nt var (i.e. the largest group of identical BcR IG nt sequences within a clonotype) of a given clonotype is identified. This represents the central point of the network since it comprises the largest fraction of sequences and, in turn, B cells of the respective clone. By default, SHMs of the main nt var are calculated from the beginning of FR1 to the end of FR3. However, this analysis can be performed from a more downstream position within the BcR IG, and suggestions are given when selecting the parameters.

Second, nt vars that have the same SHMs as the main nt var as well as additional SHMs are selected. Whenever the main nt var does not carry any SHM (i.e. 100% germline identity), all other nt vars will be assigned to that category. Samples that do not contain nt vars with additional SHMs or if they contain only nt vars with a single additional SHM (one level of extra SHMs) will not be further analyzed.

Third, nt vars are connected according to their SHMs. This process takes place only if the connection is consistent with progressive acquisition of SHMs. The tool also allows for the inclusion of ‘jumps’ (i.e. connection of nt vars with common SHMs differing by two or more SHMs). Additionally, a filter regarding the minimum size of analyzed nt vars can be applied (default option is 10 sequences). In complex cases where a given nt var could be connected either to a nt var with only one SHM difference or one with jumps (difference of two or more SHMs), only the first connection would be shown for simplification purposes.

The last part of this process concerns the selection of nt vars with common yet fewer SHMs than the main nt var. These nt vars are considered as having emerged prior to the main nt var. Then, they are ordered according to their number of SHMs. At this step, all nt vars with fewer SHMs compared to the main nt var while also passing the threshold of the minimum number of sequences are considered. The reason for this additional criterion is that these nt vars are extremely rare, given that they represent early steps of the ongoing SHM process.

### Mutational pathways selection

**Figure 2:**
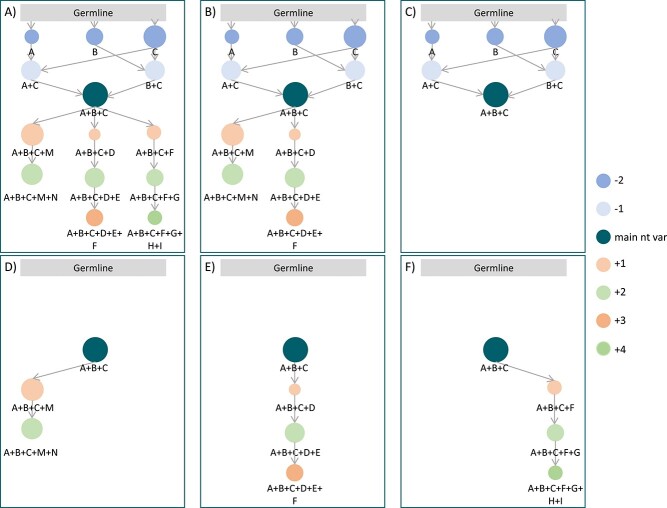
ID network and pathway selection. Representation of the complete networks and its components. The nodes represent nt variants (nt vars) and the edges represent the connections among them. The letters below the nt vars represent the mutations at the nt level that each nt var has. Note that the nt mutations are shown in this figure for the purpose of this example; they do not appear in the actual graphs, only the mutations at the aa level are shown, if any. The germline is represented in this figure for explanation purposes, but it does not appear in the actual graphs. The main nt is indicated at the center of the networks in a dark circle with three mutations (A–C). Toward it, nt vars lacking some of those mutations appear (the darker the circle, the less mutations they have and the closer to the germline they are). From the main nt var, nt vars having the same mutations (A–C) and additional ones are shown in circles orange and green (the colors are intercalated in order to distinguish better the different level of mutations; the darker the color, the higher is the number of additional mutations). The size of the nodes is proportional to the number of reads that constitutes the nt var. Panels **A** and **B** represent complete networks, the first allowing jumps (from +2 mutations to +4) and the second, without jumps allowed. As it can be seen in panel **A**, if jumps are allowed and a nt var (A + B + C + D + E + F) can be connected to the previous one (A + B + C + D + E) but also to another one with jumps (A + B + C + F), it will only be connected to the one with less difference in terms of number of mutations to simplify the graph. Panels **C**–**F** represent the pathways that are taken into account for the network construction. **C** represents the ‘less mutations pathway’ (the pathway that connects the nt vars between the main nt var and the germline). **D** represents the most relevant pathway (the one that gathers the maximal number of reads). **E** represents the longest pathway (length = 3). **F** represents the longest mutational pathway (+4 mutations). For simplicity, the three types of pathways displayed only contain one pathway each. It is, however, possible for each type to have more than one pathway (i.e. many pathways with the same maximal pathway length or with the same maximal mutational pathway length).

After the generation of the graph network, only the nt vars with SHMs considered as critical parts of the ID process within a given BcR IG clonotype are selected. In more detail, as previously mentioned, all nt vars with fewer SHMs than the main nt var are selected. In regard to nt vars with additional SHMs, only the nt vars belonging to the following pathways are selected for further analysis (Figure 2):

(i) Most relevant pathway. Also named as the main block of SHMs, it is defined as the group of nt vars leading to a specific end of pathway nt var (end node) and including the highest number of BcR IG sequences within a given clonotype.
(ii) Longest pathway(s). They contain the highest number of nodes.
(iii) Longest mutational pathway(s). They include connected nodes with the highest cumulative number of mutations. It is identical to the ‘longest pathway’ if ‘jumps’ are not allowed.

### Graph metrics calculations

High levels of complexity in the ID reflect a strong selective antigenic pressure, leading to concrete, specific and non-random mutational patterns. To obtain an overview of such process, we introduced a series of graph network metrics. They can be used to quantify different features related to the complexity of the ID process, such as the level of convergence or the length of the mutational pathways (Table 1). In brief, a sample with a high level of complexity in the ID process would be characterized by high ‘average degree’, ‘average distance’, ‘relative reads convergence’ score, ‘maximal path length’ and ‘maximal mutational length’ as well as by a low ‘end nodes density’ score. The metrics ‘average degree’ and ‘average distance’ are established Graph Theory metrics [45]. The rest (‘relative reads convergence’, ‘end nodes density’, ‘maximal path length’ and ‘maximal mutational length’) have been specifically designed for ID analysis.

**Table 1:** Description of the graph metrics used for the characterization and comparison of ID

Metric	Calculation	Meaning	High value =	Question that is answered by the metric	
RELATIVE READS CONVERGENCE	$$\frac{\begin{array}{c}\mathrm{Number}\ \mathrm{of}\ \mathrm{sequences}\ \mathrm{of}\\{}\ \mathrm{the}\ \mathrm{most}\ \mathrm{relevant}\ \mathrm{pathways}\end{array}}{\begin{array}{c}\mathrm{Number}\ \mathrm{of}\ \mathrm{sequences}\ \mathrm{of}\\{}\ \mathrm{the}\ \mathrm{main}\ \mathrm{nt} \operatorname{var}\end{array}}$$	Tendency for the BcR IG sequences to accumulate in the main nt var or to acquire additional convergent SHMs	High convergence	Will the clone evolve toward the accumulation of more SHMs or will it remain in its current state?	
END NODES DENSITY	$$\displaystyle\frac{\mathrm{Number}\ \mathrm{of}\ \mathrm{end}\ \mathrm{nodes}}{\begin{array}{c}\mathrm{Number}\ \mathrm{of}\ \mathrm{nt}\ \mathrm{vars}\ \mathrm{with}\ \\{}\mathrm{additional}\ \\{}\ \mathrm{SHMs}\end{array}}$$	Randomness or specificity of the mutational path	High randomness	Is the acquisition of SHMs specific or random?	
MAXIMAL PATH LENGTH	Number of levels of additional SHMs	Complexity of the mutational path	High complexity	Is this an ongoing SHM process happening in many steps?	
MAXIMAL MUTATIONAL LENGTH	Maximum level of additional SHMs	Complexity of the mutational pathway, allowing non-consecutive SHMs	High complexity	Are there many different SHMs happening progressively?	
AVERAGE DEGREE	Average total number of connections of each nt var	Complexity and connectivity of the mutational pathways	High connectivity	Are the mutations strongly interconnected?	
AVERAGE DISTANCE	Average number of steps along the shortest pathways between each pair of nt vars	Complexity of the mutational path	High complexity	Is the ID process complex?	

In the case of the relative reads convergence, a low value would be close to 0, with the possibility of having cases with values >1 (if the nt vars with additional mutations accumulate more BcR IG sequences than the main nt var). For the end nodes density, the closer to 0, the lower the randomness of the ID process. For the rest of the proposed metrics, there cannot be a single defined range of values that can be readily considered as ‘low’ or ‘high’. In principle, the further a value is away from 0, the greater the level of the complexity is for the respective metric. Ultimately, the comparison between samples across multiple metrics can allow for better estimation of the ID complexity evaluation.

### Identification of replacement SHMs

For each node, SHMs at the nt level are classified into silent and replacement SHMs, which lead to amino acid (aa) substitutions. This process is performed for the main nt var as well as for all nt vars with additional SHMs.

### Graph network visualization

For graph network visualization, the main nt var is selected and depicted as the central point. Nodes that represent nt vars with fewer SHMs are depicted with directionality toward the main nt var according to the connectivity pattern; in specific, the greater its SHM difference, the further away it is depicted. For the nt vars with additional SHMs, only nodes and edges that fall into the previously mentioned pathways are selected and represented with directionality from the main nt var. Depending on the selected parameters, the network representation may also contain: (i) aa substitutions (this does not include the SHMs of the main nt var in order to better visualize the acquisition of new replacement SHMs); (ii) the relative size of each individual nt var can be proportional to its number of sequences using a log10 scale and (iii) SHMs jumps (if allowed, nodes with non-consecutive SHMs will be shown).

The main nt var is always depicted in dark petrol blue, whereas the nt var with fewer SHMs are represented using different shades of lighter blue (the darker the shade, the greater the distances from the main nt var). For the nt vars with additional SHMs, different shades of orange and green are intercalated, allowing for a rapid distinction of mutational levels and the identification of non-consecutive SHMs (nt vars with ‘jumps’). Jumps can be visually identified whenever two connected nodes have the same color and/or there is a gap in intensity between their respective shades of colors (the darker the code, the greater the distance from the main nt var).

### Group comparisons

Information regarding different groups and their respective samples should be provided by the user. Then, all groups are compared for each individual metric. First, a Kruskal-Wallis test is applied in order to determine whether the comparisons are overall statistically significant [46]. Subsequently, pairwise comparisons are performed for all samples and the statistical significance of each comparison is calculated with the Wilcoxon test [47]. The user can select whether the *p*-values will be adjusted or not. For a given metric, the corresponding values of each group are represented with side-by-side boxplots. The *x*-axis corresponds to the groups, while the *y*-axis corresponds to the metrics values. Furthermore, the plot contains the mean/median value as a red dot/horizontal line within the boxplot of each group. Whenever a pairwise comparison between two groups is statistically significant, the plot will also contain a bar connecting these two groups as well as the corresponding *p*-value directly above.

### The dataset

The dataset used for the validation of IgIDivA was extracted from a previous NGS study focusing on the ID analysis in chronic lymphocytic leukemia (CLL) samples from stereotyped subsets #2 and #169 [31, 48]. The dataset is publicly available in the repository European Nucleotide Archive (ENA) with the accession number PRJEB36589.

In more specific, the dataset consisted of 72 analyzed PCR amplicons from 44 patients with CLL: 32 cases belonged to stereotyped subset #2 (32 IGHV-IGHD-IGHJ, 21 IGLV-IGLJ gene rearrangements), 7 cases belonged to stereotyped subset #169 (7 IGHV-IGHD-IGHJ, 6 IGLV-IGLJ gene rearrangements), while 6 cases were not stereotyped (6 IGLV-IGLJ gene rearrangements).

## Results and discussion

### General output

The output of IgIDivA consists of a series of graphs and summary tables in order to provide a systematic analysis of the level of ID. In more specific terms, the following types of output are provided for each given sample:

(i) Summary calculations table. It contains information about the number of clonotypes that are considered for analysis, the number of nt vars, the number of total sequences, the number of singletons, the number of expanded sequences and the number of sequences of the main nt var.
(ii) Extra mutations calculations. It provides the number of nt vars with additional SHMs for each given number of SHMs as well as the total number of sequences. It includes the total number of nt vars and sequences (i.e. not only the ones that are plotted and used for the calculation of graph metrics).
(iii) Fewer SHMs calculations. This table shows the number of sequences lacking SHMs of the main nt var for each different number of SHMs.
(iv) Evolution. It provides information for all unique SHMs or combinations of SHMs of all the nt vars that are part of the connected graph network. It also shows the number of SHMs in comparison to the germline, the number of sequences with those SHMs and the mutational level to which they belong. In more detail, the mutational level is ‘less’ if they have fewer SHMs than the main nt var, ‘main’ for the SHMs of the main nt var and ‘additional’ for the cases with more SHMs than the main nt var. This information may assist in the identification of SHMs that are represented in a given network at the nt level and their connectivity pattern.
(v) Block table. This table contains the following information for each end node: (i) the block score (i.e. the ratio of the total number of sequences of the nodes forming the block of pathways that leads to that particular end node to the total number of sequences of all the nodes of the network with more SHMs than the main nt var); (ii) convergence score (i.e. relative reads convergence metric, Table 1); (iii) number of sequences of the block of pathways; (iv) number of nodes of the block of pathways; (v) id of nodes forming the block of pathways; (vi) pathways length and (vii) maximal mutational length of the block. From this table the group of nodes with the highest block score is selected as the ‘main block’ or the ‘most relevant pathways’.
(vi) Graph network. It contains all features described in the ‘Network generation’ subsection of the Materials and Methods section.
(vii) Graph info. It summarizes the values of the graph metric. It contains the germline identity %, the values of the graph metrics (Table 1) as well as information related to those metrics.
(viii) Replacement mutations. It consists of two different tables with the information of the replacement SHMs in the main nt var and the rest of the nt vars, respectively. Information for all SHMs at the aa level and the number of sequences carrying each mutation is provided. These changes might have implications at the functional level.

When analyzing more than one sample, additional output is produced:

(i) Discarded samples table. It provides the names of samples that have been discarded from the analysis (e.g. samples with no connections among nt vars).
(ii) aa mutation tables. It contains all identified replacement SHMs, including those present in more than one sample.
(iii) Metrics table. It includes the graph metrics information for all samples. If a sample has been discarded, the cause is provided.
(iv) Comparisons. If samples are classified into groups, the tool performs pairwise comparisons for all groups. This is performed independently for each of the graph metrics.

### Application: ID assessment in CLL stereotyped subsets #2 and #169

To assess the capacity of IgIDivA to provide a systematic and multifaceted analysis of ID, we processed the data from the study by Gemenetzi *et al*. [31, 48], concerning the analysis of samples from patients with CLL belonging to stereotyped subsets #2 and #169.

In CLL, ‘stereotyped subsets’ are defined as groups of patients presenting common immunogenetic features as well as common clinical presentation and outcome [49–52]. Previous studies have shown that stereotyped subset #2, the largest in CLL, displays pronounced ID within the IG heavy and (lambda) light chain genes [31, 53]. Stereotyped subset #169 is considered a satellite to subset #2, i.e. it is closely related at the immunogenetic level [54]. Gemenetzi *et al*. explored this relationship through the study of ID within the BcR IG utilizing NGS [31]. The authors reported a median of 3662 nt variants for the dominant heavy chain gene clonotype of subset #2 cases and 1803 variants/dominant clonotype for subset #169 cases (*p*-value = 0.479). Regarding the light chain clonotypes, a median of 7015 variants/dominant clonotype was reported for subset #2 cases, while the respective value for subset #169 was 5101 variants (*p*-value = 1.000). The non-subset cases displayed lower ID levels for the dominant clonotype (*p*-value < 0.05) compared to subset #2, but no statistically significant differences were found against subset #169. Despite these findings, the analysis was not able to capture the full extent of the ID process. Thus, we decided to analyze the ID process in these two stereotyped subsets in CLL in a more systematic way.

We followed the same pre-processing of data as outlined in the original study by Gemenetzi *et al*. [31] and then proceeded to the dedicated ID analysis with IgIDivA. Because of differences in the experimental approach for the PCR amplification and NGS of the heavy and light chain sequences, the starting point of the alignment had to be different for the samples with IGHV-IGHD-IGHJ gene rearrangements and the ones with IGLV-IGLJ gene rearrangements (column start = 5 and 23, respectively). Thus, samples were divided in two different batches and analyzed with IgIDivA (Table 2).

**Table 2:** Number of samples and running time of the two different batches of samples analyzed with IgIDivA

IG gene rearrangements	Number of samples	Running time	Running time/sample
IGHV-IGHD-IGHJ	39	3 min 20 s	5.13 s
IGLV-IGLJ	33	3 min 49 s	6.9 s

Some of the samples were discarded from the analysis, as described in Table 3.

**Table 3:** Number of samples discarded from the analysis and number of samples remaining for the analysis

Subset	Rearrangements	Discarded (maximal mutational length = 1)	Discarded (no connected nodes with extra SHMs)	Remaining for the analysis
#2	IGHV-IGHD-IGHJ	12	3	17
#2	IGLV-IGLJ	5	0	16
#169	IGHV-IGHD-IGHJ	0	0	7
#169	IGLV-IGLJ	1	0	5
Non-subset	IGLV-IGLJ	1	0	5

**Figure 3:**
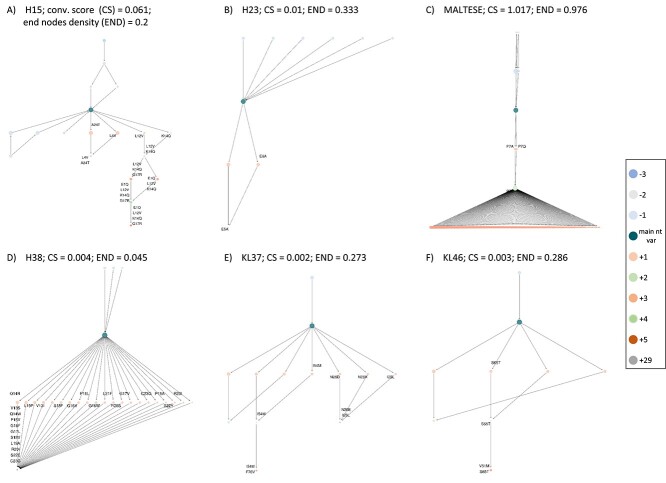
Different levels of complexity and convergence in the ID process can be depicted with the IgIDivA graph networks. Panels **A**–**C** are subset #2 CLL samples (**A** and **B** are samples with IGHV-IGHD-IGHJ gene rearrangements, while **C** is a sample with IGLV-IGLJ rearrangement), **D** and **E** are subset #169 samples (IGHV-IGHD-IGHJ and IGLV-IGLJ gene rearrangements, respectively) and **F** is a non-subset sample (IGLV-IGLJ gene rearrangement). All networks have different levels of complexity and convergence of the ID (e.g. **A** has the longest pathways, **B** has only three nodes with additional mutations, **C** has many (121) end nodes, while **D** has only one end node and **E** and **F** have three and two end nodes, respectively). The figure shows the need for graph metrics to measure and quantify these characteristics and the differences or similarities among groups. For a better understanding, the values of the metrics convergence score and end nodes density of each sample has been added next to their respective identifier. The sample with the highest convergence score (**C**, CS = 1.017, indicating a higher accumulation of the BcR IG sequences in the nt vars with additional mutations compared to the main nt var), is also the sample with the highest END (indicating a higher level of end nodes and, thus, a higher level of randomness). This is an example of how it is necessary to have several metrics in order to correctly characterize one sample. **A** has the second highest convergence score, with a low END, showing a high level of convergence and complexity of the mutational pathways in the ID process.

Finally, comparisons were undertaken between the different groups for all metrics (running time = 6.5 s). In total, all the analysis was performed in 7 min 26 s, with an average of 6.05 s/sample. The analysis was performed using a standard mid-range laptop, with two processor cores (Intel Core i3) and 4 GB RAM.

**Figure 4:**
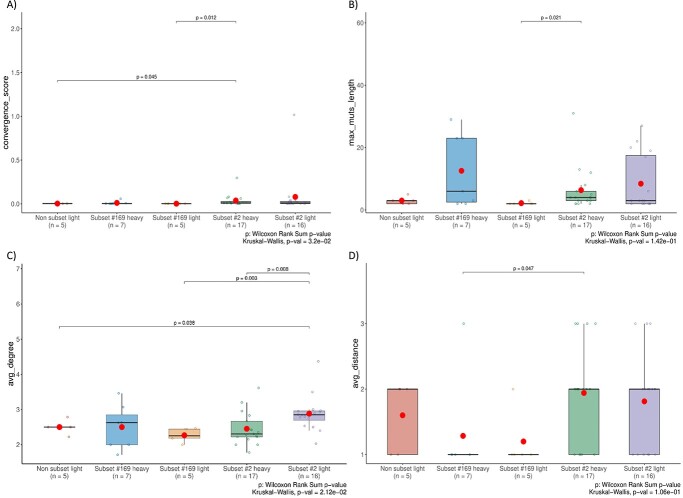
Some of the pairwise comparisons between the sample groups displayed statistically significant results. Each set of boxplots represents a different graph metric (**A**, convergence score; **B**, maximal mutational length; **C**, average degree; **D**, average distance). Pairwise comparisons were performed for each metric among the groups (non-subset IGLV, subset #169 IGHV, subset #169 IGLV; subset #2 IGHV and subset #2 IGLV). The values are shown as boxplots, with each boxplot being a group, the *y*-axis representing the value of the metric, the horizontal line in the middle of the boxplot representing the median and the dot inside the boxplot representing the mean. If a pairwise comparison is statistically significant, the *p*-value is shown. For each metric, the *p*-value of the overall Kruskal Wallis comparison is shown as well. ‘Light’ = light chain, IGLV-IGLJ rearrangement; heavy = heavy chain, IGHV-IGHD-IGHJ rearrangement.

Evidence of ID was found in all three groups (Figure 3). Overall, subset #2 cases displayed a higher level of complexity in the ID process, followed by #169 cases and non-subset cases. In most cases, the most prominent differences concerned the IGLV-IGLJ data. For example, in terms of convergence score, the IGL sequences of subset #2 had mean/median values of 0.079/0.0105, followed by IGH sequences of subset #2 cases (mean/median 0.038/0.01), IGH subset #169 sequences (mean/median 0.011/0.003), non-subset IGL sequences (mean/median 0.003/0.003) and, finally, subset #169 IGL sequences (mean/median 0.002/0.002). Nevertheless, differences between the groups were small and only a few pairwise comparisons displayed statistically significant differences (Figure 4):

(i) Convergence score: Subset #2 IGH sequences versus non-subset IGL sequences (mean/median 0.038/0.010 and 0.003/0.003, respectively; *p*-value = 0.045); subset #2 IGH sequences versus subset #169 IGL sequences (mean/median 0.038/0.01 and 0.002/0.002, respectively; *p*-value = 0.012).
(ii) Maximal mutational length: Subset #2 IGH sequences versus subset #169 IGL sequences (mean/median 6.35/4 and 2.2/2, respectively; *p*-value = 0.021).
(iii) Average degree: Subset #2 IGL sequences versus subset #169 IGL sequences (mean/median 2.881/2.850 and 2.268/2.250, respectively; *p*-value = 0.003); subset #2 IGL sequences versus subset #2 IGH sequences (mean/median 2.881/2.850 and 2.45/2.31, respectively; *p*-value = 0.008); subset #2 IGL sequences versus non-subset IGL sequences (mean/median 2.881/2.850 and 2.502/2.500, respectively; *p*-value = 0.038).
(iv) Average distance: Subset #2 IGH sequences versus subset #169 IGH sequences (mean/median 1.94/2 and 1.286/1, respectively; *p*-value = 0.047).

All *p*-values were calculated using the Wilcoxon test [47] in order to evaluate the statistical significance of changes in the metrics’ distribution medians between the provided groups. The other metrics and pairwise comparisons did not show any statistically significant differences. This highlights the overall high level of similarity between the analyzed groups and, perhaps most importantly, the ability of IgIDivA to identify even small differences in terms of ID that otherwise could remain unseen.

### IgIDivA versus other bioinformatic tools

As previously mentioned, IgIDivA was designed in order to overcome limitations of previous bioinformatic tools [32–40]. First, IgIDivA does not perform any kind of inference for missing nt var connections and non-consecutive related mutations are allowed due to their potential relevance. This is particularly important considering that inferred nt variants could lead to an artificially higher level of complexity, especially in cases with extensive ID. Also, IgIDivA selects certain pathways to display in the graph (see Mutational pathways selection) leading to the generation of ‘cleaner’ graphs containing only relevant information. Moreover, IgIDivA is flexible since a variable number of clonotypes can be analyzed, depending on the user needs. Also, IgIDivA is publicly available and easy-to-use without requiring a certain level of programming skills from the user.

For the comparison of IgIDivA with an already existing bioinformatic tool, we chose Alakazam since it is a complete solution for the study of the adaptive immune receptor (IG and T cell receptor) gene repertoire and is publicly available. Alakazam has functionalities related to aa substitutions and graph metrics calculation and has been already used for the assessment of ID [30]. In terms of functionality, the tool offers the capacity to study clonal lineages, diversity analysis, gene usage and lineage reconstruction [38]. This comparison between Alakazam and IgIDivA may provide some insights regarding the corresponding strengths and challenges. To this end, we selected one sample from the previously analyzed dataset (H33) and proceeded to a similar type of analysis with both tools. Given the fact that the individual functionalities of both tools are quite different, a direct, quantitative comparison was not feasible; yet, some qualitative differences could be identified.

**Figure 5:**
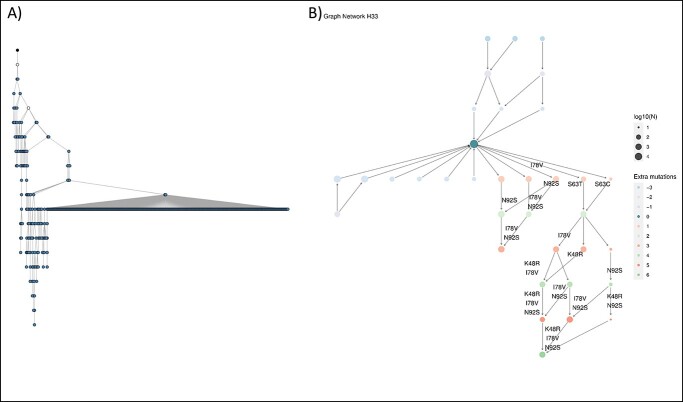
Graphs obtained with Alakazam and IgIDivA. Sample H33 analyzed with (**A**) Alakazam and (**B**) IgIDivA. IgIDivA provides a more detailed characterization of the nt vars that are part of the ID process, including information of their size, their SHM content and the mutations at the aa level. Alakazam displays inferred nt vars (uncoloured circles) and depicts all the nt vars in the sample (coloured circles). IgIDivA does not infer intermediate nt vars but allows the presence of ‘jumps’ (two nt vars can be connected while having more than one SHM of difference) and the nt vars that are represented are the ones that are part of the ‘less mutational pathway’, the ‘most relevant pathway’, the ‘longest pathway’ and the ‘longest mutational pathway’ (i.e. the pathways that contribute the most to the ID process). Consequently, the results are more focused on the ID analysis and provide more meaningful results in that regard.

First, the input was modified in order to enable processing it with Alakazam. The sample was analyzed with IMGT/HighV-QUEST and the output was extracted in AIRR format. Second, a script with a combination of some of the functionalities of Alakazam had to be written due to the fact that this tool is not specialized for ID analysis and it has a wider range of uses: (i) makeChangeoClone and (ii) buildPhylipLineage. Then, a lineage tree was obtained, which was compared to the network obtained with IgIDivA (Figure 5).

Some of the differences found between the two methods were the following:

(i) Processing time: Alakazam took approximately 3 min to provide the output, while IgIDivA’s output was produced in 10 s.
(ii) Presence of inferred nt vars: Alakazam includes in its lineage tree inferred nt vars together with all the nt vars present in the data. In contrast, IgIDivA focuses only on real nt vars included in pathways directly related to the ID process.
(iii) aa mutations: Alakazam offers the possibility to study the aa content of sequences and its properties. However, it is not possible to integrate this information into the network. IgIDivA plots the aa substitutions in the networks and provides tables with all the aa mutations present in all the samples.
(iv) Graph metrics: Alakazam provides the option of calculating some metrics, however, most of them are calculated individually for each nt var. For example, the function ‘getPathLengths’ calculates the distance from the germline for each nt var; the ‘summarizeSubtrees’ function gives as result a table with some properties of each node (such as the size, the pathway length or the outdegree). While Alakazam provides comparisons between samples of the distributions of normalized subtree statistics for a population of trees with the ‘plotSubtrees’ option, it is not possible to get the metrics results per sample (instead of per node) or to make comparisons between groups of samples. IgIDivA’s metrics are specific to the ID analysis, and comparisons between groups of samples can be performed.
(v) Purpose-built end-to-end tool: Unlike Alakazam, IgIDivA has been designed specifically for the analysis of ID; thus, it offers a complete workflow that requires a minimal effort from the user’s side.
(vi) User-friendly tool: IgIDivA is also an easy-to-use Shiny app without any need for programming knowledge. Moreover, the scripts are available and can be modified if necessary.

Overall, Alakazam offers many useful functionalities for a complete repertoire analysis. However, IgIDivA is more specific in the context of the ID process and provides a complete view in that regard.

## Conclusions

The study of ID in different contexts, extending from infections and vaccination to autoimmunity, allergy and B cell malignancy, can assist in gaining insight into the implicated processes, with diagnostic, prognostic and therapeutic relevance. Hence, dedicated tools can meaningfully assist in thorough immunogenetic analysis; against that, however, already available tools are not always specific for this purpose, have some drawbacks or do not provide the necessary detailed characterization. For these reasons, we developed IgIDivA, a purpose-built and publicly available tool for the complete and detailed analysis of the ID process in high-throughput sequencing data. IgIDivA offers several functionalities: (i) retrieval of different types of immunogenetic information, (ii) identification of SHMs and establishment of mutational pathways, (iii) visualization of graph networks representing SHM connections involved in the ID process, (iv) calculation of new graph-based metrics that were developed for the evaluation of ID levels and (v) application of statistical analysis for the assessment of comparisons between groups of samples. Moreover, IgIDivA provides summary tables throughout the analysis that the user can use for downstream study, while, at the same time, it is fast, open-source and easy-to-use for both experienced and inexperienced users. To our knowledge, IgIDivA is the first tool developed specifically for the detailed study and characterization of the ID process, offering relevant information, visual representations and useful metrics for quantification together with statistical analysis for the comparison among samples. It can be applied for the study of ID in many different contexts and samples.

Future steps regarding the development of IgIDivA include compatibility to the AIRR format as well as support of additional visualizations of the output. Moreover, the flexibility of IgIDivA offers the possibility of further future extensions, including more graph metrics, additional parameters or continuous customization.

**Key Points**

- The availability of bioinformatic tools tailored for the analysis of ID is limited.
- We present IgIDivA, a purpose-built tool that provides a detailed characterization and visualization of the ID process, allowing for comparisons between samples or groups of samples accompanied by advanced statistical analysis.
- IgIDivA can provide relevant information about the ID process in infections, vaccination’s reactions, autoimmunity, allergy and B cell malignancies, among others, possibly having implications in their diagnosis, prognosis and treatment.
- IgIDivA is fast, publicly available and user-friendly and available also as a Shiny app web application.

## Data Availability

The data underlying this article are available in the repository ENA with the accession number PRJEB36589, at https://www.ebi.ac.uk/ena/browser/view/PRJEB36589.

## Notes

**Laura Zaragoza-Infante** is a PhD student of immunogenetics at the Institute of Applied Biosciences (INAB), at the Centre for Research and Technology Hellas (CERTH) and is associated with the Faculty of Medicine at the Aristotle University of Thessaloniki. Her research interests include the immunogenetic study of B cell lymphomas and the application of bioinformatics in immunogenetics.

**Dr Valentin Junet** was, at the time of this study, employed by Anaxomics Biotech S.L. and was a PhD candidate at the Autonomous University of Barcelona. His research interests include bioinformatics and the application of machine learning to biomedical data.

**Nikos Pechlivanis** works as a bioinformatics engineer at INAB|CERTH. He is pursuing a PhD in the field of computation biology and bioinformatics. His research focuses on studying machine learning algorithms and computational techniques with respect to their corresponding applications on genomic data.

**Styliani-Christina Fragkouli** obtained her BSc in physics from the Aristotle University of Thessaloniki in 2019 and is working as research assistant at INAB|CERTH. Her interests include machine learning and bioinformatics.

**Serovpe Amprachamian** is currently an undergraduate student of electrical and computer engineering at the Aristotle University of Thessaloniki. His research interests include software development, machine learning, deep learning and their for data analysis and mining in life and medical sciences.

**Dr Triantafyllia Koletsa** is an associate professor of pathology at the School of Medicine, Aristotle University of Thessaloniki. Her research interests focus on tumor biomarkers, the immunogenicity of a tumor and its microenvironment.

**Dr Anastasia Chatzidimitriou** is a senior researcher at INAB|CERTH. Her research activities focus on the field of immunobiology and genomics applying NGS methodologies for the detection of new biomarkers in cancer.

**Dr Maria Papaioannou** is a professor of hematology at the Aristotle University of Thessaloniki and is a collaborating researcher at INAB|CERTH. Beyond her clinical work, her research interest focuses on immunogenetics of hematologic diseases (ALL, HIV Lymphomas, CLL and PNH).

**Dr Kostas Stamatopoulos** is a specialist in hematology, director of INAB|CERTH. His research interests include precision medicine in cancer, immunobiology of lymphoid malignancies and patient empowerment in care.

**Dr Andreas Agathangelidis** is an assistant professor of molecular genetics at the Department of Biology of the National and Kapodistrian University of Athens. His research interests concern the characterization of genes, signaling pathways and cellular mechanisms associated with the ontogeny and pathogenesis of lymphoid malignancies.

**Dr Fotis Psomopoulos** is a researcher at INAB|CERTH. His research interests lie at the intersection of bioinformatics and machine learning, primarily working on the design and implementation of novel algorithms for knowledge extraction from large datasets in life sciences.
